# Supplementary material for: Resolving the full spectrum of human genome variation using Linked-Reads
Source: Genome Res. 2019 Apr;29(4):635–45. doi: 10.1101/gr.234443.118 (PMC6442396; doi:10.1101/gr.234443.118)
Supplement: Supplemental Material [file supp_gr.234443.118_Supplemental_longranger-2.2.zip › longranger-2.2.tar.bz2!/lr2.2-public/lib/stan/cmdstan/stan/doc/api/html/stanc__helper_8hpp.html]

Stan: src/stan/command/stanc\_helper.hpp File Reference


|  |  |
| --- | --- |
|  | Stan  2.5.0  probability, sampling & optimization |


- Main Page
- Related Pages
- Namespaces
- Classes
- Files

- File List
- File Members

All Classes Namespaces Files Functions Variables Typedefs Enumerations Enumerator Friends Macros Pages

- src
- stan
- command

Functions

stanc\_helper.hpp File Reference

`#include <stan/version.hpp>`  
`#include <stan/gm/compiler.hpp>`  
`#include <exception>`  
`#include <fstream>`  
`#include <iostream>`  
`#include <stdexcept>`  
`#include <string>`  
`#include <stan/io/cmd_line.hpp>`

Go to the source code of this file.

|  |  |
| --- | --- |
| Functions | |
| void | print\_version (std::ostream \*out\_stream) |
|  | |
| void | print\_stanc\_help (std::ostream \*out\_stream) |
|  | Prints the Stan compiler (stanc) help. More... |
|  | |
| void | delete\_file (std::ostream \*err\_stream, const std::string &file\_name) |
|  | |
| int | stanc\_helper (int argc, const char \*argv[], std::ostream \*out\_stream, std::ostream \*err\_stream) |
|  | |

## Function Documentation

|  |  |  |  |
| --- | --- | --- | --- |
| void delete\_file | ( | std::ostream \* | *err\_stream*, |
|  |  | const std::string & | *file\_name* |
|  | ) |  |  |

Definition at line 55 of file stanc\_helper.hpp.

|  |  |  |  |  |  |
| --- | --- | --- | --- | --- | --- |
| void print\_stanc\_help | ( | std::ostream \* | *out\_stream* | ) |  |

Prints the Stan compiler (stanc) help.

Definition at line 26 of file stanc\_helper.hpp.

|  |  |  |  |  |  |
| --- | --- | --- | --- | --- | --- |
| void print\_version | ( | std::ostream \* | *out\_stream* | ) |  |

Definition at line 12 of file stanc\_helper.hpp.

|  |  |  |  |
| --- | --- | --- | --- |
| int stanc\_helper | ( | int | *argc*, |
|  |  | const char \* | *argv*[], |
|  |  | std::ostream \* | *out\_stream*, |
|  |  | std::ostream \* | *err\_stream* |
|  | ) |  |  |

Definition at line 65 of file stanc\_helper.hpp.


---

[ Stan Home Page ]

*© 2011–2014,
Stan Development Team.*
